# Neurocritical progression in amyotrophic lateral sclerosis: pathological relevance and validation

**DOI:** 10.1515/biol-2025-1323

**Published:** 2026-05-14

**Authors:** Jiayue Pan, Chengyi Zhang, Jingyao Li, Li Ma

**Affiliations:** School of Medicine, Wuhan University of Science and Technology, Wuhan, Hubei Province, China

**Keywords:** amyotrophic lateral sclerosis, neurocritical care, respiratory failure, advanced-stage state, multisystem dysfunction, pharmacological management

## Abstract

Evidence from multiple clinical studies indicates that amyotrophic lateral sclerosis (ALS) frequently evolves into a condition requiring neurocritical care. In advanced stages or during acute complications, ALS can rapidly transition into a neurocritical state characterized by respiratory insufficiency, systemic dysfunction, and accelerated neurological decline. Although current management strategies for advanced-stage ALS are relatively well established, there remains a significant lack of targeted interventions aimed at preventing or attenuating neurocritical deterioration. This review systematically examines the pathophysiological mechanisms underlying neurocritical progression in ALS, including respiratory failure, metabolic imbalance, autonomic dysfunction, and multisystem involvement. We further evaluate emerging and potential therapeutic strategies designed to mitigate disease severity and stabilize critical neurological function. In addition, we analyze clinical and biological factors that increase susceptibility to neurocritical states and discuss evidence-based approaches to delay disease progression. By integrating clinical observations with mechanistic insights, this review aims to improve early recognition, optimize neurocritical management, and ultimately enhance outcomes for patients with ALS.

## 1 Introduction

Amyotrophic lateral sclerosis (ALS), also known as Lou Gehrig’s disease, is a progressive and fatal neurodegenerative disorder characterized by the selective degeneration of upper and lower motor neurons, leading to muscle weakness, paralysis, and ultimately death [1]. Although ALS typically follows a gradually progressive course, a substantial subset of patients, particularly in advanced stages, experience acute and life-threatening complications, including respiratory failure, severe dysphagia, and systemic infections, which can precipitate a neurocritical state [2], 3].

In ALS, a neurocritical state refers to a period of physiological instability marked by compromised vital functions, most commonly due to respiratory insufficiency, profound swallowing dysfunction, or multisystem failure. These conditions frequently necessitate intensive interventions such as invasive or non-invasive mechanical ventilation, enteral or parenteral nutritional support, and comprehensive neurointensive monitoring [4], 5]. Unlike classical neurocritical illnesses characterized by impaired consciousness or coma, many patients with end-stage ALS remain cognitively intact. and do not develop classic Nevertheless, mortality most often results from respiratory failure, malnutrition, or systemic infection rather than primary loss of consciousness [6]. Management of ALS during this neurocritical phase therefore presents unique clinical and ethical challenges and requires specialized, multidisciplinary care [7]. Despite its clinical importance, systematic investigation of neurocritical deterioration in ALS remains limited.

From a pathogenetic perspective, recent advances in ALS genetics have substantially expanded our understanding of disease heterogeneity and mechanisms underlying rapid clinical decline. In addition to well-characterized pathogenic mutations in *SOD1* and *C9orf72*, large-scale genomic studies have identified novel ALS-associated variants in genes such as *NEK1* and *KIF5A* [8], 9], among others. These discoveries implicate a complex pathogenic network involving disrupted RNA metabolism, impaired axonal transport, defective DNA damage repair, and loss of proteostatic control. Mutations in *SOD1*, *C9orf72*, *NEK1*, and *KIF5A* have been associated with accelerated respiratory muscle degeneration and an increased susceptibility to neurocritical complications. Moreover, converging evidence suggests that neuroinflammatory cascades, mitochondrial dysfunction, and bioenergetic failure further exacerbate rapid neurological and systemic deterioration, highlighting the urgent need for targeted interventions during neurocritical progression [10].

From a clinical standpoint, this review first examines the pathophysiological mechanisms driving neurocritical deterioration in ALS, with particular emphasis on respiratory failure, bulbar dysfunction, metabolic imbalance, and secondary systemic complications. We then evaluate current neurocritical strategies for ALS, including mechanical ventilation modalities, nutritional and metabolic support, infection control, and the management of cognitive and psychological symptoms. Finally, we discuss emerging therapeutic approaches, such as gene-based therapies, stem cell strategies, and novel immunomodulatory agents, and highlight key gaps in knowledge that must be addressed to improve survival, functional outcomes, and quality of life for patients with ALS in neurocritical settings.

A comprehensive literature search was conducted to identify relevant studies published up to April 2025. Databases including PubMed, Web of Science, Embase and Google Scholar were searched using combinations of the following terms: “amyotrophic lateral sclerosis,” “ALS,” “neurocritical care,” “neurointensive care,” “respiratory failure,” “mechanical ventilation,” “sepsis,” “dysphagia,” “palliative care,” and “end-stage.” Reference lists of included articles were manually screened to identify additional relevant publications.

Priority was given to: (1) clinical trials, particularly randomized controlled trials (RCTs), and large observational cohort studies involving ALS populations; (2) systematic reviews and meta-analyses; (3) seminal preclinical studies elucidating mechanisms directly relevant to neurocritical deterioration. When evidence from non-ALS critical care populations is cited, it is identified as such, and conclusions drawn from these data are interpreted cautiously, emphasizing the need for validation in ALS-specific studies.

## 2 Pathogenesis of ALS in neurocritical conditions

### 2.1 Genetic basis of ALS in neurocritical conditions

The transition of ALS from a chronic neurodegenerative disorder to a neurocritical condition is highly heterogeneous and strongly influenced by genetic backgrounds. Specific pathogenic variants are associated with accelerated disease progression, early involvement of bulbar and respiratory musculature, and an increased susceptibility to acute, life-threatening complications that necessitate neurocritical care. Elucidating these genetic determinants is essential for identifying patients at elevated risk of rapid deterioration and for developing anticipatory and personalized management strategies.

Mutations in the *superoxide dismutase 1* (*SOD1*) gene account for a significant subset of familial ALS and are frequently associated with a rapidly progressive, predominantly limb-onset phenotype that often culminates in early respiratory failure. At the molecular level, mutant SOD1 proteins undergo misfolding and aggregation within motor neurons, leading to oxidative stress and progressive neurodegeneration [11]. Extensive experimental evidence implicates mitochondrial dysfunction [12], endoplasmic reticulum stress [13], and impaired protein homeostasis [14] as major contributors to this process. Whether through direct effects on mitochondrial proteins or indirect disruption of cellular bioenergetics, SOD1-associated ALS is characterized by reduced ATP production and excessive reactive oxygen species (ROS) generation [15]. During periods of acute physiological stress, such as infection or respiratory compromise, energetically impaired mitochondria are unable to meet increased metabolic demands, resulting in rapid neuronal dysfunction. Motor neurons innervating respiratory muscles are particularly vulnerable due to their continuous high metabolic activity, thereby accelerating respiratory muscle weakness and precipitating respiratory failure.

**Figure 1:**
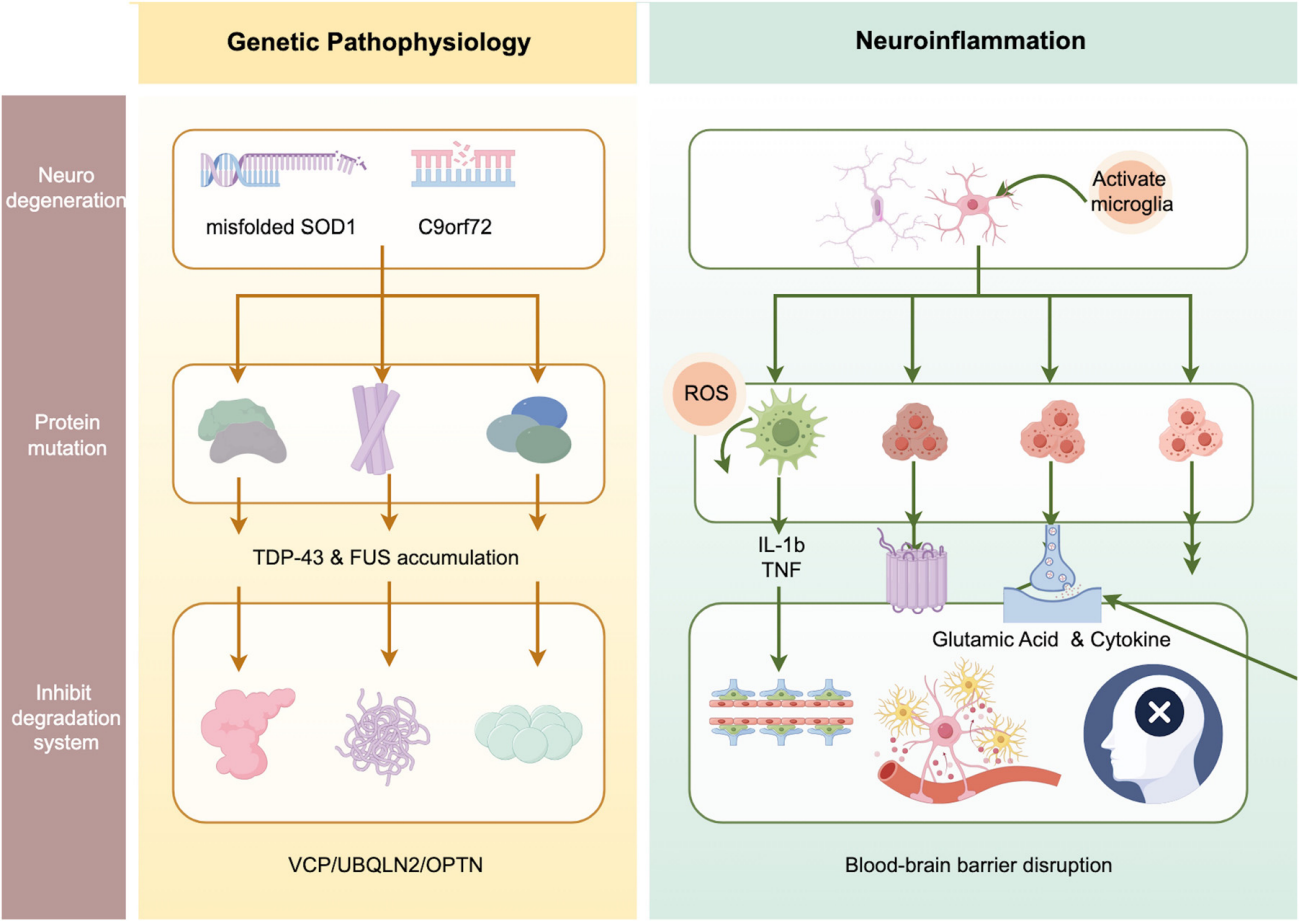
Development of ALS in neurocritical care. Genetic and inflammatory mechanisms driving neurocritical deterioration in ALS. This schematic illustrates how ALS-associated genetic mutations and neuroinflammatory cascades converge to accelerate respiratory motor neuron vulnerability and precipitate neurocritical states. Left panel (genetic pathophysiology): ALS-related genetic alterations, including misfolded SOD1 and C9orf72 repeat expansions, lead to abnormal protein aggregation and impaired RNA processing, culminating in TDP-43 and FUS accumulation. Disruption of protein degradation pathways involving VCP, UBQLN2, and OPTN further exacerbates cellular stress, reducing neuronal resilience under acute physiological challenges. Right panel (neuroinflammation): Genetic and proteostatic stress activates microglia, promoting the release of pro-inflammatory cytokines (e.g., IL-1β, TNF) and reactive oxygen species. These processes enhance glutamate-mediated excitotoxicity and compromise blood–brain barrier integrity, amplifying susceptibility to acute respiratory failure and neurocritical deterioration.

Epidemiological studies indicate that hexanucleotide repeat expansion in the *C9orf72* gene represents the most common genetic cause of familial ALS. This mutation consists of an abnormal expansion of the GGGGCC repeat sequence within a noncoding region of gene [16], leading to RNA-mediated toxicity and the production of toxic dipeptide repeat proteins accumulate within neurons and glial cells, disrupting nucleocytoplasmic transport, proteostasis, and synaptic function [17]. Clinically, *C9orf72*-associated ALS frequently overlaps with frontotemporal cognitive and behavioral impairment, which may compromise patients’ capacity to participate in decision-making regarding life-sustaining interventions such as mechanical ventilation or gastrostomy. In addition, autonomic dysfunction observed in this genetic subtype may contribute to acute cardiovascular instability during critical illness. At the pathological level, RNA toxicity and dipeptide repeat accumulation provoke robust neuroinflammatory responses, with microglial activation playing a key role in driving neuronal injury and systemic deterioration [18] (Figure 1) (Table 1).

**Table 1:** ALS genetic subtypes with implications for neurocritical care management.

Gene	Typical disease course	Neurocritical care relevance	ICU implication	References
*C9orf72*	Rapid progression, ALS-FTD overlap	Bulbar/behavioral impairment, decision-making difficulty	Early goals-of-care/sedation caution	[16], 19]
*SOD1*	Variable, some aggressive	Higher likelihood of ventilator dependence	Early NIV → planning invasive ventilation	[20], 21]
*TARDBP*	Faster functional decline	Early bulbar & respiratory involvement	Early swallowing & airway protection	[22], [23], [24]
*FUS*	Early-onset, aggressive	Prone to earlier respiratory decompensation	Close respiratory monitoring	[25], [26], [27]
*SQSTM1*	Rare, autophagy/FTD overlap	System involvement (protein clearance)	Early ICU referral (conservative)	[28], 29]
*UBQLN2*	ALS + dementia phenotype	Poor compliance due to cognitive/behavioral problems	Family-centered decision-making	[10]
*VCP*	MSP1 (multi-system)	Muscle + system weakness	Multidisciplinary ICU planning	[30]

a: Mutation frequency and mechanism. Mutations in the *C9orf72* gene (repeated amplification of intron GGGGCC) account for approximately 40 % of familial ALS cases and cause neuroinflammation through RNA toxicity and accumulation of dipeptide repeat proteins (DPR). b: Pathological influence. *SOD1* mutations lead to protein misfolding, induce oxidative stress and mitochondrial dysfunction, and are common in familial ALS. c: RNA metabolism defect. *TARDBP* mutations disrupt RNA splicing and stability, leading to TDP-43 cytoplasmic aggregation, which is associated with ALS and frontotemporal dementia (FTD). d: Clinical phenotype. FUS mutations are common in early-onset ALS, with symptoms including cognitive decline (FTD comorbidities), and the mechanism involves disorders in nucleoplasmic transport. e: Degradation pathway failed. Mutations in *SQSTM1* impair autophagy function and accelerate abnormal protein accumulation, which are more common in sporadic ALS cases. f: X linkage inheritance. The *UBQLN2* mutation is X-linked dominant, disrupting the ubiquitin-proteasome system and leading to dementia characteristic. g: Multi-system effects. VCP mutations affect nuclear membrane integrity and autophagy, and are often clinically combined with bone Paget’s disease and cognitive impairment.

Mutations in *TARDBP*, which encodes TAR DNA-binding protein 43 (TDP-43) represent another major genetic contributor to ALS [31]. Abnormal cytoplasmic aggregation of TDP-43 is observed in the vast majority of ALS cases, including both familial and sporadic forms, as well as in frontotemporal lobar degeneration (FTLD) [32]. Similarly, mutations in the *Fused in Sarcoma* (*FUS*) gene, although less prevalent than *SOD1* or *C9orf72* mutations, lead to nuclear-to-cytoplasmic mislocalization of the FUS protein and subsequent formation of toxic intracellular aggregates [33]. Pathological TDP-43 and FUS disrupt multiple aspects of RNA metabolism, including splicing, transport, and local translation, ultimately compromising neuromuscular integrity. These disruptions disproportionately affect bulbar and respiratory motor neurons, thereby shortening the interval between symptom onset and neurocritical deterioration [34].

### 2.2 Involvement of neuroinflammation and motor neuron degeneration in neurocritical conditions

In neurocritical settings, neuroinflammation is responsible for precipitating and amplifying acute neurological deterioration in patients with ALS. Accumulating evidence suggests that inflammatory activation within the central nervous system (CNS) lowers the physiological threshold for respiratory failure, increases motor neuron vulnerability, and compromises the brain’s capacity to adapt to acute systemic stressors such as infection, hypoxia, and metabolic imbalance [35]. As a result, even transient inflammatory insults may trigger disproportionate neurological decline in patients with advanced ALS.

Beyond the CNS, the gastrointestinal tract has emerged as a critical regulator of systemic inflammation and a key contributor to multisystem dysfunction in critically ill patients [36].

Disruption of gut barrier integrity and alterations in the intestinal microbiota can promote translocation of proinflammatory mediators and pathogens into the systemic circulation, thereby exacerbating systemic inflammatory responses [37]. In ALS, this inflammatory milieu may increase susceptibility to sepsis and multiple-organ dysfunction syndrome (MODS), both of which are major determinants of mortality in neurocritical care settings [38]. Acute infections and systemic inflammatory insults represent particularly potent triggers of neurocritical deterioration in ALS. Supporting the role of systemic inflammation, a clinical study by Tortelli et al. demonstrated significantly elevated plasma levels of inflammatory cytokines, including IL-6 and TNF-α, in patients with ALS [39]. Although these observational findings do not establish a direct causal relationship, they indicate a higher basal inflammatory state that may predispose patients to rapid clinical decline when exposed to acute stressors.

Importantly, the relationship between inflammation, infection, and ALS appears to be bidirectional. Systemic infections can exacerbate neuroinflammation and potentially accelerate motor neuron neurodegeneration through sustained activation of CNS immune pathways. Conversely, ALS patients are highly susceptible to infections due to bulbar dysfunction, impaired cough and airway clearance, respiratory muscle weakness, and frequent exposure to invasive supportive measures such as mechanical ventilation and feeding tubes [40]. This bidirectional relationship between infection and inflammation is particularly relevant in neurocritical care settings.

Taken together, neuroinflammation in ALS represents a multifaceted and self-perpetuating process involving microglial and astrocytic dysfunction, oxidative stress, glutamate-mediated excitotoxicity, and disruption of the blood–brain barrier (BBB). These interconnected mechanisms form a vicious cycle linking systemic inflammatory responses to central motor neuron degeneration, thereby accelerating disease progression and increasing vulnerability to neurocritical complications [41] (Figure 1).

### 2.3 Systemic effects of ALS-related respiratory failure leading to neurocritical conditions

Respiratory failure represents one of the most severe and debilitating complications in patients with ALS and is a principal driver of neurocritical deterioration. Progressive degeneration of motor neurons innervating the diaphragm [42], intercostal muscles [43], and accessory respiratory musculature [44] leads to a gradual decline in ventilatory capacity. As respiratory muscle strength diminishes, effective ventilation becomes increasingly compromised, resulting in impaired gas exchange and reduced respiratory reserve. In the early stages of respiratory involvement, patients may develop exertional dyspnea, orthopnea, or nocturnal hypoventilation. As the disease progresses, inadequate ventilation becomes more pronounced, placing patients at high risk of chronic and acute hypoventilation, defined by insufficient alveolar ventilation to meet metabolic demands [45]. Hypoventilation leads to hypercapnia [46], characterized by elevated carbon dioxide (CO_2_) levels [45], and is frequently accompanied by hypoxemia. Together, these abnormalities significantly impair oxygen delivery to neural tissue and accelerate neuronal injury. Evidence from critical care and experimental neuroscience demonstrates that sustained hypoxemia and hypercapnia induce neuronal energy failure, glutamate-mediated excitotoxicity, mitochondrial dysfunction, and oxidative stress [47]. Prolonged cerebral hypoxia disrupts synaptic transmission, impairs neuronal survival pathways, and amplifies neuroinflammatory responses, thereby increasing the risk of acute neurological manifestations such as altered consciousness, seizures, and encephalopathy [48]. Furthermore, hypoxemia and hypercapnia promote BBB permeability, facilitating the entry of peripheral inflammatory mediators into the CNS and further exacerbating neuroinflammation. Systemic inflammatory activation accelerates these pathological cascades, contributing to autonomic instability and diffuse cerebral dysfunction [47]. Collectively, these processes often necessitate urgent neurocritical care intervention to prevent irreversible neurological decline.

**Figure 2:**
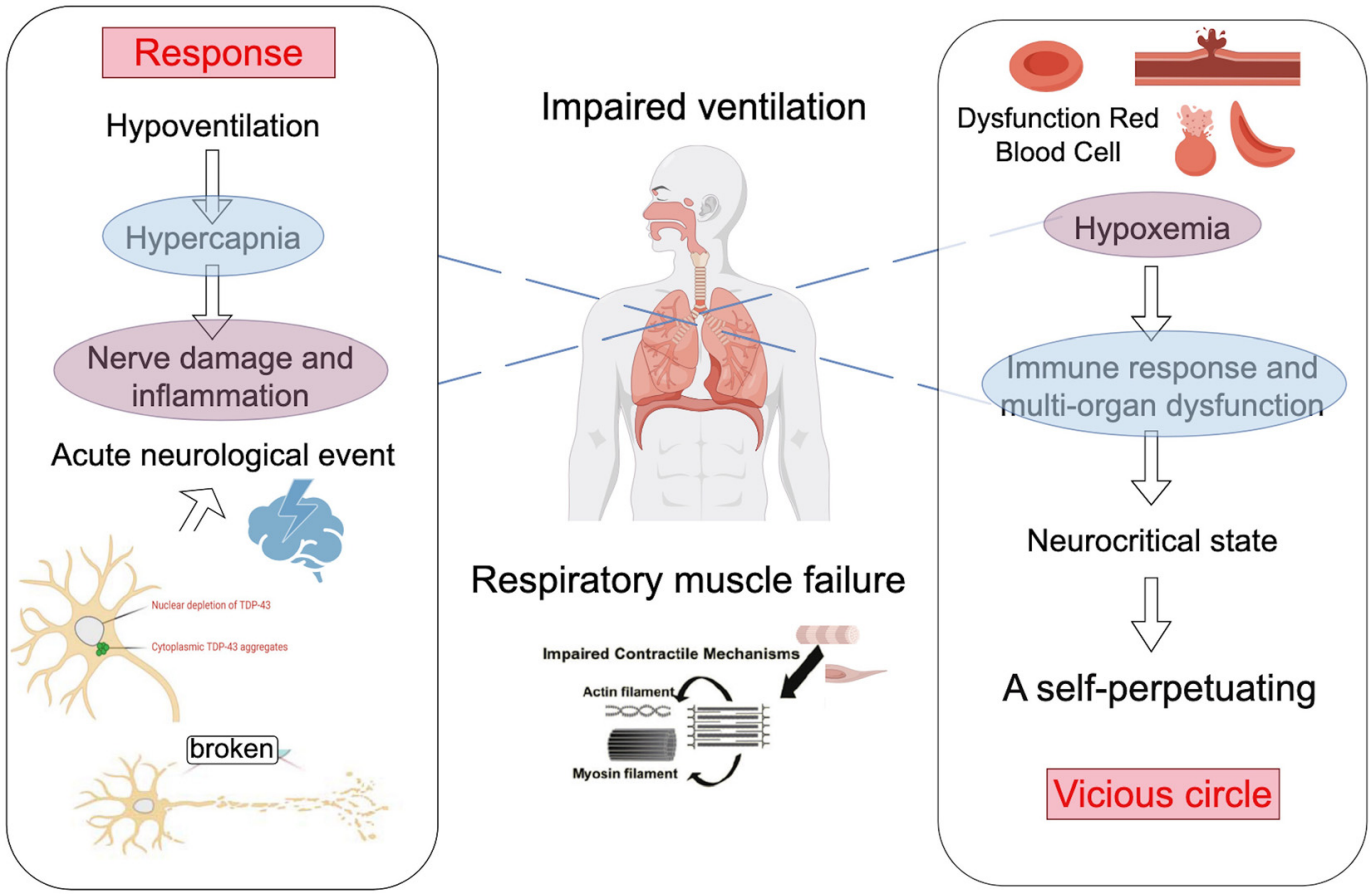
The systemic effects of ALS-related respiratory failure on neurocritical conditions. ALS progression leads to respiratory muscle weakness, impaired ventilation, hypoventilation, hypercapnia, hypoxemia, and neuroinflammation. The clinical outcomes include neurological complications, like seizures, multi-organ dysfunction affecting the cardiovascular, renal, and gastrointestinal systems, and the need for intensive neurocritical care. The interaction between these two sections highlights how respiratory failure can trigger a self-perpetuating cycle of systemic and neurological decline in ALS, underscoring the need for comprehensive management.

Respiratory failure also serves as a potent trigger for systemic complications, including infection, sepsis, and multiorgan dysfunction [48]. Recurrent aspiration, impaired airway clearance, and prolonged ventilatory support increase susceptibility to pulmonary infections, while hypoventilation-related acidosis and immune dysregulation further compromise host defenses. Concurrently, hypercapnia, hypoxemia, and ventilatory insufficiency accelerate the transition of ALS into a neurocritical state, necessitating intensive respiratory and neurological support [49]. Beyond the CNS, respiratory failure exerts widespread systemic effects that indirectly worsens neurological outcomes. Hypoxia and systemic inflammation impair cardiovascular function [50], reduce renal perfusion and clearance [51], and disrupt gastrointestinal barrier integrity, leading to metabolic derangements and endotoxemia [52]. These multisystem disturbances destabilize cerebral homeostasis and predispose patients to secondary neurological complications, including septic encephalopathy, cerebral ischemic events, and severe autonomic dysregulation (Figure 2).

Taken together, ALS-related respiratory failure initiates a self-reinforcing cycle of hypoventilation, systemic inflammation, multiorgan dysfunction, and central nervous system injury. This cascade highlights the critical importance of early recognition, continuous monitoring, and aggressive management of respiratory failure to mitigate neurocritical complications and improve outcomes in patients with ALS.

## 3 Clinical management of ALS in neurocritical settings

Progression of ALS to a neurocritical state is not inevitable but rather represents a culmination of distinct, often sequential complications. Proactive, multidisciplinary management aimed at preventing or attenuating these complications constitutes the foundation of effective neurocritical care in ALS. The primary goals are to: (1) delay the onset of life-threatening organ dysfunction, particularly respiratory failure); (2) prevent acute secondary insults, such as aspiration pneumonia, sepsis, and metabolic decompensation that can precipitate abrupt neurological decline; and (3) mitigate the physiological and neurological consequences of critical events when they occur. The following sections outline evidence-based strategies across these domains, together forming a comprehensive clinical framework for reducing the risk of neurocritical deterioration in ALS.

### 3.1 Respiratory failure and ventilatory support

Progressive degeneration of motor neurons in ALS leads to continuous weakening of respiratory muscles, especially the diaphragm and intercostal musculature. This decline in respiratory muscle strength results in hypoventilation and impaired gas exchange, leading to hypercapnia and hypoxia. These abnormalities can cause respiratory acidosis, impaired cognition, daytime somnolence, and rapid deterioration of respiratory function, ultimately necessitating urgent intervention [53]. Therefore, timely and effective ventilatory support is crucial in managing respiratory failure among patients with ALS. In the early to intermediate stages of respiratory compromise, non-invasive ventilation (NIV) is the preferred treatment option for patients with mild-to-moderate ventilatory insufficiency [54]. NIV is most commonly delivered via bilevel positive airway pressure (BiPAP), which provides higher inspiratory pressure to augment ventilation and lower expiratory pressure to facilitate exhalation [55]. By reducing the work of breathing and improving alveolar ventilation, NIV effectively mitigates carbon dioxide retention and alleviates symptoms of hypoventilation [56]. Clinical trials have demonstrated that NIV is particularly beneficial during sleep, when hypoventilation is often most pronounced, and is also effective for managing daytime respiratory insufficiency. Reported adherence rates approach 85 % in those with early-stage respitaory failure [57]. Moreover, NIV use has been associated with reduced hospitalization rates, prolonged survival in selected populations, and marked improvements in quality of life [5]. Its non-invasive nature and relative tolerability make NIV an optimal strategy during earlier stages of respiratory decline. However, its effectiveness diminishes as respiratory muscle weakness advances, particularly in the presence of severe bulbar dysfunction, poor mask tolerance, or progressive dyspnea.

As ALS progresses and respiratory muscles can no longer sustain adequate ventilation, invasive mechanical ventilation may become necessary [58]. Tracheostomy is typically considered in advanced respiratory failure when long-term ventilatory support is required [59]. This intervention enables more reliable airway access and continuous ventilatory assistance when diaphragmatic and intercostal muscle function is profoundly compromised. Although invasive ventilation can provide continuous support, it also presents significant challenges, including the risk of ventilator-associated pneumonia [60], recurrent infection progressing to sepsis, airway stenosis and tracheal injury [61]. Retrospective analyses indicate that prolonged dependence on invasive mechanical ventilation demands intensive caregiving, may significantly affect patients’ perceived quality of life, and increases the risk of secondary complications such as respiratory muscle atrophy, pulmonary injury, and systemic deconditioning [62]. These factors must be weighed carefully when choosing the most appropriate treatment measure.

**Figure 3:**
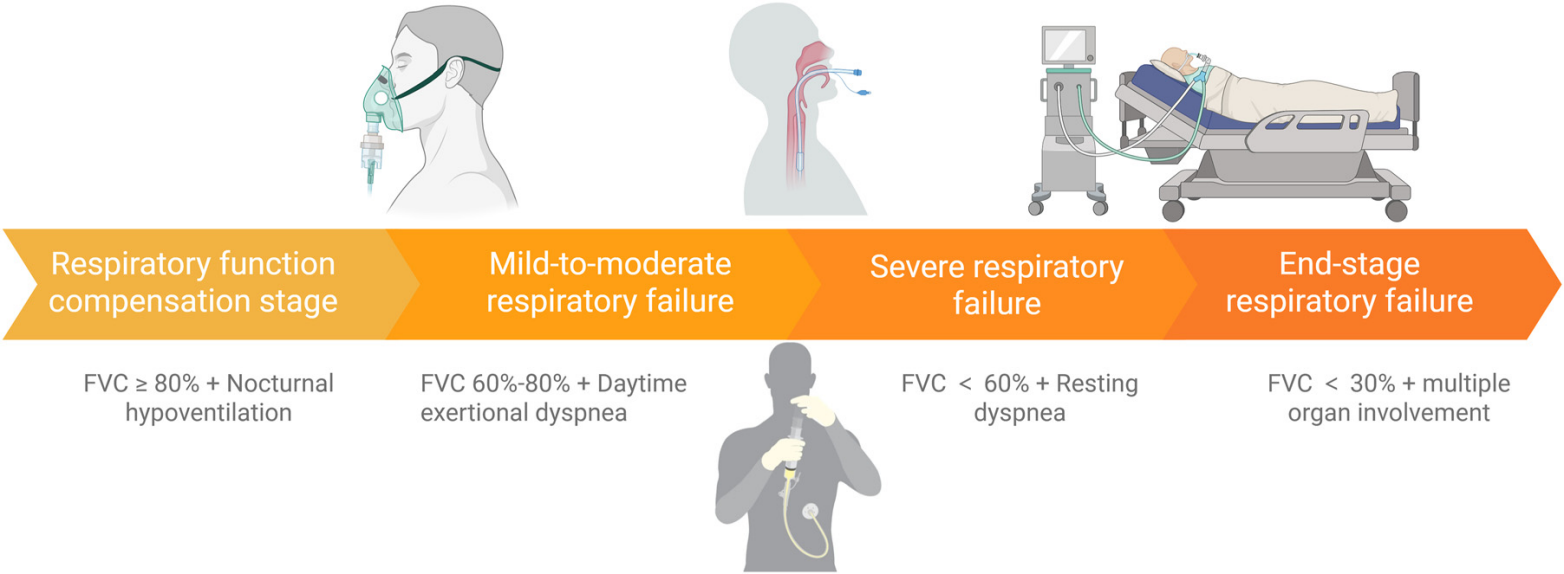
Neurocritical progression trajectory in amyotrophic lateral sclerosis (ALS). The schematic summarizes four clinically relevant phases: Pre-neurocritical phase, impending neurocritical transition, established neurocritical state, and post-critical trajectory. It links early respiratory and bulbar vulnerability to acute triggers, including aspiration, infection, secretion burden, hypercapnia, and nutritional deterioration, and highlights major neurocritical manifestations and decision nodes relevant to NIV, PEG, intubation or tracheostomy, and goals-of-care planning.

Figure 3 illustrates the neurocritical progression trajectory of ALS, linking early respiratory and bulbar vulnerability to acute decompensation triggers, established neurocritical manifestations, and downstream care pathways. Rather than depicting respiratory decline alone, the revised figure emphasizes clinically actionable decision nodes, including escalation to preventive NIV, consideration of PEG before nutritional collapse, discussion of intubation or tracheostomy during acute deterioration, and goals-of-care or family-centered decision-making in the post-critical phase.

### 3.2 Swallowing dysfunction and nutritional support

Swallowing dysfunction (dysphagia) is a common and clinically significant complication of ALS, particularly in patients with bulbar involvement [63]. Progressive degeneration of motor neurons innervating the pharyngeal, laryngeal, and tongue musculature leads to impaired coordination of the oral and pharyngeal phases of swallowing. Clinically, this manifests as difficulty initiating swallowing, poor bolus control, delayed airway closure, and ineffective clearance of food and liquids, substantially increasing the risk of choking and aspiration [64]. Aspiration of oropharyngeal contents into the lower airway is a major contributor to aspiration pneumonia, which remains one of the leading causes of morbidity and mortality in ALS [65]. In neurocritical settings, aspiration pneumonia frequently serves as a precipitating event for acute respiratory failure, sepsis, and intensive care unit (ICU) admission, thereby accelerating neurocritical deterioration. Early identification of swallowing impairment is therefore critical. Instrumental assessments, including video-fluoroscopic swallowing study (VFSS) and fiberoptic endoscopic evaluation of swallowing (FEES), may help detect clinically important dysphagia before overt decompensation occurs [66].

Loss of effective swallowing function also imposes major nutritional and respiratory risks. Many patients with ALS experience progressive weight loss, dehydration, and reduced oral intake due to prolonged mealtimes, fear of choking, and impaired swallowing safety [67]. In this context, nutritional support is relevant not only for long-term maintenance but also for prevention of neurocritical transition. Enteral nutritional support, most commonly via percutaneous endoscopic gastrostomy (PEG), is recommended for patients with moderate to severe dysphagia, recurrent aspiration, or clinically significant weight loss [68], [69], [70]. In neurocritical care settings, PEG is particularly valuable for reducing recurrent aspiration events, preserving nutritional stability during ventilatory support, and facilitating medication administration. Regular monitoring of body weight, hydration status, and swallowing safety is therefore essential, as timely PEG placement may reduce the risk of aspiration-related deterioration and help stabilize patients before respiratory or systemic crises develop [71], [72], [73].

### 3.3 Cognitive and psychological complications

Beyond progressive motor impairment, ALS is frequently accompanied by substantial cognitive and psychological disturbances that significantly influence disease trajectory and clinical management. Depression and anxiety are highly prevalent among patients with ALS, reflecting the profound emotional burden of living with a progressive, life-limiting illness and the cumulative loss of functional independence [74]. These psychological symptoms often intensify as the disease advances, further diminishing quality of life and complicating engagement with complex medical care. In addition, Beeldman et al. [75] demonstrated that ALS is not confined to motor neuron pathology but frequently involves frontotemporal brain regions responsible for executive function, behavior, and decision-making [76]. This cognitive-behavioral spectrum ranges from mild executive dysfunction to overt frontotemporal dementia (FTD), suggesting the concept of ALS as a multisystem neurodegenerative disorder. In neurocritical care settings, cognitive impairment and psychological distress directly compromise patients’ capacity to participate in informed decision-making, communicate effectively with care teams, and adhere to life-sustaining interventions such as mechanical ventilation or enteral feeding [77], [78], [79]. Psychological interventions, such as cognitive behavioral therapy (CBT), can improve depression, anxiety, and maladaptive thought patterns, ultimately enhancing their ability to participate in the care program [80]. In neurocritical care, where stress, sensory deprivation, and loss of autonomy are common, the psychological burden of ALS is further amplified. This necessitates an integrative, patient-centered treatment approach that extends beyond traditional neurological management [81], 82]. A recent scoping review highlights the importance of embedding cognitive and psychological care within neurocritical management frameworks [83]. Optimal care therefore requires a multidisciplinary team comprising neurologists, psychologists, psychiatrists, speech-language pathologists, and social workers. Such teams are essential for addressing the complex interplay between cognitive impairment, emotional distress, ethical decision-making, and intensive medical interventions [84]. Integrating psychological and cognitive support into neurocritical care not only improves adherence to treatment and communication but also mitigates the psychological burden associated with prolonged critical illness.

### 3.4 Pharmacological management

Current pharmacological therapies for ALS are primarily intended to modestly slow disease progression or relieve selected symptoms, rather than rescue patients from established neurocritical deterioration. The neurocritical relevance of these agents lies less in reversing ICU-stage respiratory failure than in modulating upstream processes that contribute to neuroinflammation, oxidative stress, excitotoxicity, and vulnerability to hypoxic-hypercapnic injury. Accordingly, their clinical value is generally preventive or trajectory-modifying rather than rescue-oriented once patients have entered an acute neurocritical state [85], 86].

Riluzole, the earliest approved disease-modifying therapy, exerts its effects through inhibition of glutamate-mediated excitotoxicity and has been consistently shown to extend survival by approximately 2–3 months [87], 88]. However, riluzole has no established direct effect on acute hypercapnic respiratory failure, severe bulbar decompensation, or other ICU-level manifestations of neurocritical ALS [79].

Edaravone, a free radical scavenger, demonstrated a reduction in ALSFRS-R decline in a narrowly defined early-stage population with preserved forced vital capacity [89]. Its intravenous or cyclic administration, coupled with the absence of evidence supporting benefit in advanced or ICU settings, substantially limits its applicability once patients enter a neurocritical state [90].

Dextromethorphan/quinidine (DHQ) is approved for the treatment of pseudobulbar affect in ALS and provides meaningful symptomatic improvement without modifying underlying disease progression [91]. While DHQ may be continued in monitored inpatient settings, its use requires caution due to cardiac conduction effects, and it does not influence respiratory failure or survival-related outcomes [92].

Tofersen, the first antisense oligonucleotide therapy approved for SOD1-mutant ALS, represents a paradigm shift toward genotype-specific, biomarker-driven treatment. Clinical trials have demonstrated robust reductions in neurofilament light chain levels and suggest delayed clinical progression when administered early [93], 94]. Therefore, the neurocritical relevance of tofersen is best understood as preventive rather than rescue-oriented: it may delay progression toward neurocritical deterioration in genetically defined patients, but it has no established role in reversing acute ventilatory failure or other ICU-stage events [95] (Table 2).

**Table 2:** Pharmacological agents in ALS and their neurocritical relevance: anti-excitotoxic, anti-neuroinflammatory, anti-hypoxic, and anti-hypercapnic implications.

Drug	Primary mechanism	Anti-neuroinflammatory relevance	Anti-hypoxic relevance	Anti-hypercapnic relevance	Target population	Key efficacy endpoints (evidence level)	Major limitations	Key references
Riluzole	Glutamate release inhibition	Indirect, limited; reduces excitotoxic signaling that can secondarily dampen neuroinflammatory activation	No direct anti-hypoxic effect; may only indirectly reduce hypoxia-vulnerable excitotoxic injury	No established anti-hypercapnic effect	All ALS phenotypes	∼2–3 month survival extension; modest ALSFRS-R slowing (RCT, meta-analysis)	Hepatotoxicity; no acute respiratory or bulbar benefit	[87]
Edaravone	Reactive oxygen species scavenging	Moderate, indirect; suppresses ROS-linked inflammatory injury	Partial; attenuates hypoxia-related oxidative damage rather than correcting hypoxemia itself	No established anti-hypercapnic effect	Early-stage ALS (FVC ≥80 %)	∼33 % slower ALSFRS-R decline in selected population (RCT)	IV/cyclic burden; no ICU or late-stage benefit	[89]
DHQ	NMDA antagonism; sigma-1 receptor agonism	No established clinically meaningful anti-neuroinflammatory effect	No established anti-hypoxic effect	No established anti-hypercapnic effect	ALS with pseudobulbar affect	∼49 % reduction in CNS-LS score; symptomatic benefit only (RCT)	QT prolongation risk; no disease modification	[92]
Tofersen	SOD1 mRNA silencing (ASO)	Yes in SOD1-ALS; reduces mutant SOD1-driven inflammatory signaling	No direct anti-hypoxic effect	No established anti-hypercapnic effect	SOD1-mutant ALS	Significant NfL reduction; delayed clinical decline (RCT)	Intrathecal delivery; biomarker-dominant effect; no acute role	[96]

ALS, amyotrophic lateral sclerosis; ALSFRS-R, ALS functional rating scale-revised; ASO, antisense oligonucleotide; CNS-LS, center for neurologic study – lability scale; DHQ, dextromethorphan hydrobromide + quinidine sulfate; FVC, forced vital capacity; GI, gastrointestinal; HR, hazard ratio; IV, intravenous; NfL, neurofilament light chain; PBA, pseudobulbar affect; RCT, randomized controlled trial; SOD1, superoxide dismutase 1.

#### 3.4.1 Riluzole

Riluzole was the first disease-modifying therapy approved by the U.S. Food and Drug Administration for ALS and remains a foundational component of standard care. Its therapeutic effect is primarily mediated through attenuation of glutamate-mediated excitotoxicity, achieved by inhibiting presynaptic glutamate release, modulating voltage-gated sodium channels, and enhancing glutamate uptake. Through these mechanisms, riluzole reduces excitotoxic neuronal injury and modestly decelerates motor neuron degeneration. Preclinical studies and large clinical trials have consistently demonstrated a modest survival benefit across diverse ALS populations, leading to its recommendation for early initiation following diagnosis, irrespective of genetic subtype. Despite its broad applicability, riluzole has several important limitations. Its efficacy diminishes in advanced disease stages, with no demonstrable acute benefit on respiratory mechanics, bulbar function, or neuromuscular strength. Adverse effects, including fatigue, nausea, and hepatotoxicity, necessitate regular liver function monitoring and may limit tolerability in critically ill patients. Furthermore, incomplete efficacy has been observed in certain genetic subtypes, including *SOD1*-mutant ALS [97], 98].

#### 3.4.2 Edaravone

Edaravone is a potent free radical scavenger that exerts neuroprotective effects by suppressing oxidative stress and preserving cellular integrity, particularly in early-stage ALS. Mechanistically, edaravone inhibits lipid peroxidation, reduces ROS accumulation, and mitigates ROS-induced mitochondrial dysfunction, thereby supporting neuronal survival [99], 100]. Its clinical efficacy appears restricted to a narrowly defined population of patients with early disease (disease duration ≤2 years, forced vital capacity ≥80 %), with the greatest benefit observed in sporadic ALS subtypes. Phase III clinical trials (MCI186-19) demonstrated a statistically significant reduction in functional decline, with approximately a 33 % slower decrease in ALSFRS-R scores over 24 weeks compared with placebo (*p* = 0.001) [101], 102]. Subsequent analyses reported a 35 % reduction in the rate of ALSFRS-R decline (mean −5.0 versus −7.5 in placebo-treated patients), with the most pronounced effects observed in limb and respiratory function domains [103]. Although edaravone has not shown a significant survival advantage, modest improvements in functional trajectory and quality of life may delay the need for invasive respiratory or nutritional interventions. Nevertheless, edaravone’s clinical utility is constrained by several factors. Adverse effects, such as bruising, gait disturbance, and infusion-site reactions, along with the logistical burden of intravenous administration. Reduced efficacy in *FUS*- and *TDP-43*-mutant ALS further narrows its applicability [104]. Importantly, there is no supporting benefit in advanced-stage disease or in patients requiring intensive care, substantially limiting edaravone’s role once ALS progresses to a neurocritical state.

#### 3.4.3 DHQ

DHQ is approved for the treatment of pseudobulbar affect (PBA) in ALS and other neurological disorders. The combination pairs dextromethorphan, a noncompetitive NMDA receptor antagonist and sigma-1 receptor agonist, with quinidine, a cytochrome P450 2D6 inhibitor that increases dextromethorphan bioavailability [105]. Data from the STAR trial demonstrated a 49 % reduction in PBA episode frequency (*p* < 0.01) and clinically significant improvements of 8–10 points on the Center for Neurologic Study-Lability Scale (CNS-LS) [96], 106]. However, DHQ has important safety considerations. Adverse effects such as dizziness and diarrhea are common, and the risk of QT interval prolongation necessitates electrocardiographic monitoring, particularly in critically ill patients or those receiving concomitant QT-prolonging medications. DHQ is contraindicated in patients with significant cardiac conduction abnormalities or advanced hepatic impairment [107].

#### 3.4.4 Tofersen

Tofersen (Qalsody) is the first antisense oligonucleotide (ASO) therapy approved for the treatment of ALS caused by pathogenic *SOD1* mutations. It exerts its therapeutic effect by selectively binding SOD1 mRNA, thereby reducing synthesis of mutant SOD1 protein and mitigating downstream neurotoxicity [108]. Emerging real-world data from the United States and other regions suggest that tofersen not only slows disease progression but may also confer modest functional improvement in a subset of patients when administered during early disease stages [109]. Given that ALS frequently evolves into neurocritical conditions, such as respiratory failure and severe bulbar dysfunction, in later stages, tofersen represents an important advance in precision medicine with the potential to delay progression toward critical illness in genetically defined patients [110]. Phase III clinical trials demonstrated robust and sustained reductions in plasma and cerebrospinal fluid neurofilament light chain (NfL) levels, a validated biomarker of axonal injury, supporting its biological efficacy [111], [112], [113]. Functional benefits, as measured by ALSFRS-R scores, were more modest and became apparent only with extended follow-up.

### 3.5 Prevention of sepsis and recurrent infections

Patients with ALS are at increased risk of recurrent infections and sepsis, which are major contributors to neurocritical deterioration and mortality. Progressive respiratory muscle weakness, impaired cough, ineffective airway clearance, bulbar dysfunction, aspiration risk, and dependence on ventilatory or enteral support collectively increase susceptibility to lower respiratory tract infection and systemic infection [70]. In advanced disease, these factors frequently interact, so that aspiration pneumonia, secretion retention, and delayed recognition of infection can rapidly precipitate acute respiratory decompensation and ICU admission. A retrospective cohort study reported that the use of β2-adrenergic agonists was associated with a reduced incidence of sepsis in patients with ALS [38]. Proposed mechanisms include enhanced phagocytic activity of alveolar macrophages and attenuation of proinflammatory cytokine production, including TNF-α and IL-6.

A meta-analysis involving 5,242 patients evaluated the efficacy of systemic antimicrobial prophylaxis and antimicrobial-coated external ventricular drain (EVD) catheters in reducing ventriculostomy-related infections (VRI) [114]. Extended systemic antibiotic prophylaxis, defined as administration beyond 24 h and continuing for the duration of catheter placement, was associated with a significantly lower incidence of VRI compared with short-term perioperative prophylaxis alone. In addition, antimicrobial-coated EVD catheters (e.g., rifampicin- or clindamycin-impregnated devices), which deliver antimicrobial agents directly into the cerebrospinal fluid, reduced the relative risk of VRI by approximately 50 % compared with standard catheters. The combination of antimicrobial-coated catheters with prolonged systemic antibiotic therapy further reduced infection risk, with reported VRI rates as low as 0.8 %.

## 4 Emerging and future directions for the treatment of ALS in neurocritical conditions

### 4.1 Gene therapy and targeted interventions

Gene-based therapies have emerged as transformative strategies in ALS, with particular relevance to neurocritical care through their potential to prevent or delay progression toward life-threatening complications. By targeting upstream genetic drivers of rapid disease progression, these interventions aim to preserve bulbar and respiratory function, thereby reducing the likelihood, severity, and frequency of neurocritical events such as respiratory failure and prolonged ventilatory dependence.

In *SOD1*-mutant ALS, antisense oligonucleotide (ASO) therapies, especially tofersen, represent a major paradigm shift toward precision medicine. Tofersen selectively binds mutant *SOD1* mRNA, leading to its degradation and a subsequent reduction in toxic protein accumulation within motor neurons [115]. This direct molecular targeting is particularly important given that *SOD1* mutations are often associated with aggressive clinical phenotypes and early respiratory compromise. Clinical trial and biomarker data indicate that sustained reduction of mutant SOD1 protein levels is associated with decreased axonal injury and slower functional decline [115]. When initiated early, such interventions may prolong the period of independent respiration and delay the need for invasive ventilatory support, a critical inflection point in neurocritical care.

Therapeutic targeting of the *C9orf72* hexanucleotide repeat expansion presents greater biological and technical complexity but holds substantial promise for mitigating neurocritical risk. This mutation often leads to the ALS-FTD overlap syndrome, in which cognitive and behavioral impairment complicates decision-making, adherence to care, and systemic stability. Experimental strategies aimed at reducing RNA foci formation or suppressing the production of toxic dipeptide repeat proteins are needed to address both motor and non-motor manifestations of disease [16], 17].

### 4.2 Stem cell therapy

Stem cell-based therapies offer a multifaceted approach to ALS by combining neuroprotective, immunomodulatory, and potentially regenerative mechanisms. Rather than relying solely on replacement of lost motor neurons, current strategies emphasize modification of the hostile neural microenvironment that accelerates motor neuron degeneration [116].

The neuroprotective effects of stem cells are largely mediated through paracrine signaling, including the secretion of neurotrophic factors such as BDNF and GDNF [117]. These molecules enhance the survival of remaining motor neurons, promote axonal outgrowth, and support synaptic plasticity, all of which are critical for maintaining neuromuscular function. In addition, several stem cell populations, particularly mesenchymal stem cells (MSCs) [118], 119], exert potent immunomodulatory effects. MSCs can suppress microglial and astrocytic activation, which are key drivers of neuroinflammation in ALS. Activated glial cells release pro-inflammatory cytokines, ROS, and excitotoxic mediators that exacerbate motor neuron injury. By attenuating glial activation, stem cell therapies reduce the levels of inflammatory cytokines such as TNF-α and IL-1β, thereby mitigating secondary neuronal damage [120]. Through stabilization of the neural microenvironment, reduction of oxidative stress, and modulation of immune responses, stem cell-based interventions may slow neurodegeneration in patients with ALS.

Stem cell-based therapies hold considerable promise in the neurocritical care of ALS. Respiratory failure, a leading cause of neurocritical states in ALS, may particularly benefit from stem cell interventions that preserve motor neuron function in respiratory muscles. In addition, stem cells can attenuate systemic inflammation [121], thereby reducing the risk of critical complications such as infection or multi-organ dysfunction. Early clinical trials have demonstrated that both intrathecal or intravenous administration of MSCs is safe and may confer functional benefits [122], while experimental approaches involving neural stem cells (NSCs) and induced pluripotent stem cells (iPSCs) are being investigated for their potential to replace or support damaged motor neurons [123]. Respiratory failure represents a pivotal complication in ALS and is a common precipitant of neurocritical illness. Stem cell therapy may enhance respiratory muscle function through combined neuroprotective and anti-inflammatory mechanisms. In animal models, stem cell administration preserved diaphragm function and delayed the onset of respiratory failure [124]. Mechanistically, human umbilical cord-derived MSCs (hUC-MSCs) have been shown to upregulate neurotrophic factors, including insulin-like growth factor-1 (IGF-1) and brain-derived neurotrophic factor (BDNF), while promoting microglial polarization toward an anti-inflammatory M2 phenotype. Consistent with these findings, a phase I/IIa clinical trial reported that patients with ALS who received two intraspinal injections of bone marrow-derived MSCs (5 × 10^6^ cells/site) at 6-month intervals experienced a 35 % slower decline in forced vital capacity (FVC) over 12 months compared with placebo-treated patients [1], 2].

Although preclinical and early clinical data are encouraging, significant challenges remain, including efficient delivery to the CNS and long-term survival of transplanted cells [125], 126]. Ongoing research is therefore focused on optimizing stem cell-based strategies, particularly for preventing or mitigating acute and neurocritical manifestations of ALS.

### 4.3 Immunomodulatory agents

Immunomodulatory agents are important for managing neuroinflammation, a key driver of acute deterioration in ALS [127]. By regulating immune responses and suppressing maladaptive inflammatory signaling within the CNS system, these therapies have the potential to mitigate ALS-associated complications, including systemic inflammation [128], respiratory failure, and encephalopathy [129]. Ongoing clinical trials are currently investigating monoclonal antibodies and immunosuppressive strategies for ALS management [130]. These approaches can decelerate disease progression, improve the quality of life, and reduce the severity of neurocritical events in patients with ALS.

Recent advances have highlighted the therapeutic potential of immunomodulatory agents in mitigating neuroinflammation associated with ALS-related neurocritical conditions. For instance, drugs targeting microglial activation, such as colony-stimulating factor 1 receptor (CSF1R) inhibitors [131], have demonstrated efficacy in reducing neuroinflammation in preclinical models, thereby potentially lowering the risk of acute neurocritical complications [132]. Similarly, astrocyte-specific interventions, like reducing glutamate release or enhancing astrocytic neuroprotective functions, are being explored to prevent secondary neuronal injury [133]. Additional approaches focus on preserving or restoring BBB integrity through matrix metalloproteinase (MMP) inhibitors and anti-inflammatory agents, with the aim of limiting peripheral immune cell infiltration and systemic inflammatory signaling within the CNS. Despite these promising developments, a major challenge remains: achieving effective control of neuroinflammation without compromising host immune defense against infections [134], a critical consideration in neurocritical care settings where patients are particularly vulnerable to sepsis and other systemic complications.

## 5 Conclusions

The progression of ALS to a neurocritical state should not be viewed solely as an inevitable terminal phase, but rather as a potentially predictable and modifiable acute-on-chronic syndrome. Current clinical practice remains largely reactive, responding to crises as they arise. Future efforts must instead prioritize early identification of patients at high risk for neurocritical deterioration, implementation of pre-emptive interventions such as timely non-invasive ventilation and gastrostomy, and development of targeted therapies that stabilize the interconnected neuro–immune–respiratory axis. Such a proactive strategy holds promise for preventing transition to intensive care and improving outcomes for patients with ALS.
